# Moral (dis)engagement with anthropogenic climate change in online comments on newspaper articles

**DOI:** 10.1002/casp.2355

**Published:** 2018-06-13

**Authors:** Ruth Woods, Sharon Coen, Ana Fernández

**Affiliations:** ^1^ Robert Gordon University Aberdeen UK; ^2^ University of Salford Salford UK; ^3^ Canterbury Christ Church University Canterbury UK

**Keywords:** climate change, disengagement, ethics, media, moral

## Abstract

Anthropogenic climate change (ACC) is widely acknowledged to be morally significant, but little is known about everyday moralising around ACC. We addressed this gap via quantified thematic analysis of 300 online comments to British newspaper articles on ACC, drawing on Bandura's moral disengagement theory. Moral disengagement through denial of ACC was widespread. Other disengagement strategies, such as palliative comparison and diminishing agency, occurred less often. There was also some moral engagement, most often through assertions of the existence of ACC and/or its harmful effects. Moral disengagement was significantly more common in comments on right wing than left wing newspapers, whereas the opposite was true of moral engagement. Although Bandura's framework provided a useful starting point to make sense of ACC moralising, it did not capture moral concerns that extended beyond its “harm/care” remit. In particular, many “denial” comments included a “dishonesty” discourse, whereby ACC proponents were accused of deception for ulterior motives. To classify this discourse as moral disengagement obscures its engagement with a different set of moral issues around trust and honesty. We suggest that Bandura's theory represents one possible “moral landscape” around ACC and could be extended to encompass a broader range of moral concerns.

## 1 INTRODUCTION

Evidence continues to accrue for the existence of anthropogenic climate change (ACC) and its likely negative consequences for humans and other species around the globe (Intergovernmental Panel on Climate Change [IPCC], 2013, 2014). Philosophers and others have, consequently, argued that ACC is an important moral issue with respect to the values of justice (those who caused the problem are not those most likely to suffer as a result; Jamieson, 2007; Laksa, 2014) and, particularly, care (focusing on harm to humans, other species, and their environment; Gardiner, 2006; Hansen, 2010; Markowitz, 2012; Seabright, 2010). However, there is a lack of research on whether and how the public engages morally with ACC (Laksa, 2014). Such engagement certainly cannot be taken for granted, with behavioural and other changes still desperately needed to curb carbon emissions (Seto et al., 2016).

Climate change has been described as “a perfect moral storm,” involving the convergence of multiple factors that make it difficult for humans to react in an ethical way (Gardiner, 2006, p. 398), at least until mitigating action may be too late (Pidgeon, 2012). A key difficulty is that high carbon behaviours are highly valued and deeply embedded in many people's lives (Gardiner, 2006; Gifford, 2011; Sheller, 2004; Steg, 2005). This can create dissonance between moral values and behaviour for those who are concerned about ACC, yet feel unable or unwilling to change their behaviours accordingly, yielding negative emotions including guilt (Markowitz & Shariff, 2012). One solution to such dissonance is motivated moral reasoning (Markowitz & Shariff, 2012). Perhaps, the most detailed formulation of such reasoning is Bandura's theory of moral disengagement, which describes a set of psychosocial mechanisms by which people selectively disengage the moral sanctions they would otherwise apply to actions that go against their moral values (Bandura, 1999; Bandura, Barbaranelli, Caprara, & Pastorelli, 1996).

Bandura et al. (1996) argue that moral disengagement operates at four psychosocial loci. Thus, people are said to disengage from harmful actions by (a) reconstruing the act as morally defensible (the behaviour locus), (b) diminishing their agency and thus responsibility (the agency locus), (c) minimising harmful effects (the outcomes locus), and/or (d) reducing the status of the victims (the recipient locus). Although the theory was not originally devised with environmental issues in mind, Bandura (2007) argues that it can be used to explain why people engage in environmentally damaging behaviours, including those contributing to ACC (see also Opotow & Weiss, 2000; Stoll‐Kleemann, O'Riodan, & Jaeger, 2001).

Studies of people's reasoning about ACC have demonstrated that people do disengage from ACC in some of the ways outlined by Bandura (2007). For instance, in focus groups and online comments, people have expressed the view that their own actions would not make any difference to ACC (Butler, 2010), and that their nation's contribution is relatively small (Woods, Coen & Fernández, 2009), both of which may operate at Locus 2 to minimise agency and culpability. Moreover, Bandura (2007) argues that ACC denial is a type of moral disengagement, operating under the third locus concerning outcomes. Denial remains prominent in public and media discourse in several settings, including the UK (Woods et al. 2009; Woods, Fernández & Coen, 2012; Jaspal, Nerlich, & Koteyko, 2013; Koteyko, Jaspal, & Nerlich, 2013).

Although these studies suggest that Bandura's framework may be a useful way of understanding public moralising around ACC, to our knowledge, no research thus far has systematically assessed this possibility. The current study investigates whether moral disengagement strategies can be discerned in online comments made by members of the public to newspaper articles on ACC. However, because we wished to analyse all moralising around ACC, we hoped to capture any instances of moral engagement as well. Research suggests that those who are morally engaged with ACC are typically highly sensitive to its potential to cause harm. Howell and Allen (2017) found that people who were actively changing their lifestyles in order to mitigate climate change were motivated particularly by harmful effects on future and vulnerable humans, while American students who saw ACC as a moral issue articulated their concerns in terms of a duty or ability to steward and protect, and the potential to cause harm to others (Markowitz, 2012).

The act of foregrounding the harmful effects of ACC might be seen as moral engagement at the third locus of Bandura's theory, outcomes, the equivalent of the minimisation of harmful effects that characterises moral disengagement at this locus. We suggest therefore that moral engagement might usefully be construed as the opposite of disengagement at each of Bandura's loci: behaviour (by focusing on the moral significance of the issue); agency (by asserting culpability and accountability of oneself or one's group); and recipient (by valuing victims and representing them as equals). Our extended framework is summarised in Table 1. The psychological reality of this extension obviously remains uncertain, but nevertheless, we suggest that it represents a potentially useful way of classifying the discursive strategies (Edwards & Potter, 1992) that commenters might employ to construct ACC in particular, moralised, ways. In the current study, we use our extended version of Bandura's framework to analyse the moral content of online comments. Our first aim was simply to map out the engagement and disengagement strategies, which people articulate in online discussions, using Bandura's theory as a framework.

**Table 1:** An extension of Bandura et al.'s (1996) framework for moral engagement and disengagement

Locus	Moral disengagement	Moral engagement
(1) Behaviour	Transform harmful practices (or inaction) into acceptable ones, via moral justification, palliative comparison with other issues or practices or euphemistic labelling	Emphasise moral significance of harmful act/inaction
(2) Agency	Diffusion or displacement of responsibility	Assert agency and accountability of individuals or groups
(3) Outcomes	Disregard, minimise, or dispute harmful effects	Foreground harmful effects
(4) Recipient	Dehumanisation or blaming of victims	Value victims, encourage empathy and equality

One possible limitation of using Bandura's theory to understand ACC moralising is its focus on the moral issue of harm/care. This is indeed the moral concern most frequently raised with respect to ACC (Gardiner, 2006; Hansen, 2010; Markowitz, 2012), and in this sense, the theory seems to be well placed to make sense of people's moralising around ACC, especially with an “engagement” extension. However, cross‐cultural research indicates that harm/care is just one of several key moral concerns, or foundations, to which humans are sensitive, others including justice, loyalty, hierarchy, sanctity, and liberty (Haidt, 2012; Haidt & Joseph, 2008; Iyer, Koleva, Graham, Ditto, & Haidt, 2012; Shweder, Much, Mahapatra, & Park, 1997). Cultures and subcultures vary in the extent to which they recognise distinct moral foundations, with the result that their members become more sensitive and responsive to transgressions in some foundations than others (Graham, Haidt, & Nosek, 2009; Haidt & Joseph, 2008). It is possible, then, that people may moralise ACC with respect to foundations other than harm/care, and that these attempts to moralise might not be captured by Bandura's scheme. For instance, Feinberg and Willer (2013) showed that environmental concerns can be discursively framed in terms of sanctity rather than harm/care, and that when they are, they appeal more to people who are more oriented to this moral foundation. Therefore, our second aim was to assess the adequacy of Bandura's harm/care‐focused framework to make sense of the full diversity of people's moral responses to ACC.

Analyses of online comments on newspaper articles must take into consideration the political leanings of the media concerned. British newspapers are ideologically polarised, between left and right wing political persuasion. Although ACC is not as ideologically divided in the UK as in the USA (Pidgeon, 2012), ACC scepticism is far more prevalent in the British right wing media than the left (Carvalho, 2007; Carvalho & Burgess, 2005; Doulton & Brown, 2009; Painter & Gavin, 2015; Woods et al. 2012). Left wing newspapers may also tend to express more concern for potential victims of ACC beyond the UK compared with their right wing counterparts (Laksa, 2014). This differential attention to victims may be considered an example of moral engagement at the fourth locus of Bandura's theory (emphasising victims), whereas scepticism about the existence of ACC may represent moral disengagement at the third locus (outcomes).

We suggest that this overall ideological difference in sympathy toward the issue of ACC in the media may extend to readers. As yet we know relatively little about whether and how media discourse on ACC is taken up, used and transformed by the public, and online comments represent a useful resource in this respect (Woods et al. 2009; Koteyko et al., 2013). The third aim of the current study was to build on the extant literature on differences between left and right wing media in their treatment of ACC, to assess whether their readers also differ. We hypothesised that moral disengagement themes would be more prevalent in comments on conservative rather than left leaning newspapers, whereas the opposite would be true of moral engagement themes. Further, we hypothesised that moral engagement would be more common than disengagement in comments on left leaning newspapers, and that the opposite would be true in comments on the right wing newspapers. Although those who comment on such articles are not necessarily representative of readerships at large, we also make use of “recommendations” data (i.e., the frequency with which comments were endorsed by other readers) to give an approximate sense of how widespread particular views are within each readership.

We analysed online comments on three newspaper articles published in late March 2014. Each reported leaked content just prior to the release of the second part of the fifth IPCC assessment report, which focused on impacts, and thus had clear relevance to the harm/care moral foundation. Therefore, we hoped that these articles would be particularly likely to trigger moralised comments, whether in the form of engagement or disengagement.

## 2 METHOD

### 2.1 Comment selection

A search of the Lexis Nexis database was carried out including all UK national newspapers in the period February 12 to May 12, 2014, which covered the release of the second and third parts of the fifth IPCC report (IPCC, 2014). The search terms used were (climate change OR global warming) AND (IPCC OR Intergovernmental Panel on Climate Change), all categorised as “major mentions.” Once duplicates were taken into account, the search yielded 82 articles, of which 31 were available online and also had at least 100 comments each. This was narrowed down to 17 articles, which were published within a week before and after the release of the second part of the IPCC report on March 31, 2014. Of these 17 articles, only five were news articles focused on the overall content of the IPCC report; others focused on specific aspects of the IPCC or the report (such as the role of politicians in writing the report, or implications of the report for wildlife) or were opinion pieces. The five appropriate articles appeared in the Telegraph (2), Guardian, Independent, and Daily Mail. We had hoped to select four articles, representing all format (broadsheet vs. tabloid) and ideology (left vs. right wing) combinations. The Guardian article (Goldenberg, 2014) was selected as the left wing broadsheet, being widely regarded as more liberal than the Independent. The Telegraph article with the most comments (Demetrio, 2014) was selected as the right wing broadsheet, and the Daily Mail article (Zolfagharifard, 2014) as the right wing tabloid. Unfortunately, there was no equivalent left wing tabloid article available.

The three selected articles were very similar in terms of content, outlining the main points of the report and contextualising it within the scope of IPCC activities. All three included appeals from experts and signatories of the report for action to address or contain the foreseeable negative outcomes of CC, which might be seen as moral engagement at Bandura's first locus (behaviour). Moreover, all articles contained statements that coordinated action had the potential to cope with negative consequences, and all reported the potentially devastating consequences of inaction, thus emphasising Bandura's second (agency) and third (outcomes) loci, respectively. Although all three articles stressed the general consensus in the scientific community on ACC, they all quoted dissenting voices (in particular, that of Professor Richard Tol) and reminded readers of past and present controversies. However, only the Daily Mail described governments as “lobbying” for a change in the wording of the report, thus potentially opening the door to speculations around the influence of political interests on the report.

For each article, we extracted the first 100 top level comments (i.e., comments that were not replies to preceding comments) for analysis. The majority of these were written by different people. For the Daily Mail, 94 commenters wrote one comment each, and three commenters wrote two comments each, so 97 different people contributed to the dataset. For the Telegraph, 71 commenters wrote one comment each, six wrote two comments each, and four wrote three comments each. In addition, five comments were written by guests, so between 82 and 87 different people contributed to the dataset. For the Guardian, 83 commenters wrote one comment each, four wrote two comments each, and three wrote three comments each, so 90 different people contributed to the dataset. All comments were analysed similarly, in line with accepted use of thematic analysis (Braun & Clarke, 2006).

Following the British Psychological Society's (2013) guidelines on internet‐mediated research, informed consent was deemed unnecessary because the comments were in the public domain (no subscription or registration was required to access them). Moreover, commenters regularly responded publicly to one another's comments, thus demonstrating a widespread recognition that the comments were public and open to scrutiny. To ensure confidentiality, only the comments themselves were analysed, not the commenters' usernames. Comments are included in the article only for the purpose of illustration of themes and do not include any identifying details.

### 2.2 Analysis

We used an adjusted version of thematic analysis (Braun & Clarke, 2006). Comments were interpreted as discursive contributions to public discourse on ACC (Edwards & Potter, 1992), through which individual people construct an orientation to ACC dialogically (Bakhtin, 1986) thus incorporating elements of both constructionist and realist epistemologies (Braun & Clarke, 2006). Initially, broad semantic‐level themes were generated by all authors through a mixture of inductive (derived from the data) and deductive (informed by Bandura's framework) approaches. The authors discussed and agreed upon a final broad coding scheme, with separate themes for moral engagement, moral disengagement (both defined in terms of Bandura's scheme), truth claims supporting ACC, and truth claims challenging ACC. Scepticism around the existence of ACC is considered a type of moral disengagement by Bandura (2007). Its counterpart, belief in the existence of ACC is not necessarily an act of moral engagement; indeed, Bandura argues that it can be a driver of motivated moral disengagement. However, unsolicited active public assertions of the reality of ACC in response to a newspaper article articulating negative consequences of ACC are discursive actions, which, we would argue, are best construed as moral engagement. Therefore, truth claims supporting ACC were categorised as moral engagement in this study. We assigned separate codes to both supporting and challenging truth claim themes because they were so prevalent in the dataset relative to other kinds of moral (dis)engagement. A small number of other codes were developed for the purposes of a different set of analyses. All themes in any given comment were coded as nonoverlapping sections of the text. If a comment included only material, that did not fit into any themes, it was coded as “other.”

All three researchers used this broad coding scheme to code all comments individually and then discussed each comment to arrive at an agreed decision. This yielded a total of 420 broad codes distributed across the 300 comments (because some comments were coded for more than one theme). To check that no one coder was overly influential in these discussions, and that the coding scheme was sufficiently reliable, 10 comments from each newspaper were randomly selected for repeat individual blind coding, several months after the main coding period had ended. To assess interrater reliability, Cohen's kappa scores were calculated between the agreed coding and the individual blind coding of each author, with 0 used where the number of codes recorded for any comment differed. All authors' individual coding agreed substantially with the agreed coding, κ = .723, .689, and .776, all *p* < .001.

Once the first broad coding was complete, one researcher coded all comments including engagement or disengagement, into a more finely differentiated set of codes based on the loci of Bandura's framework. Approximately 20 each of the engagement and disengagement comments were second coded blind by another author to check interrater reliability, using Cohen's kappa. There was substantial agreement for moral disengagement, κ = .788, *p* < .001, and moral engagement, κ = .612, *p* < .001.

Finally, we scrutinised the data for any moral issues that had not already been captured by the codes derived from Bandura's framework. Morality was defined broadly, minimally encompassing Haidt and Joseph's (2008) foundations. We identified one moral theme, which we named “Dishonesty.” One author coded all comments for the presence or absence of this theme. To check interrater reliability, 39 comments were blind second coded by another author, and Cohen's kappa was calculated. There was substantial agreement, κ = .663, *p* < .001.

Although quantitative measures of prevalence are not an essential element of thematic analysis (Braun & Clarke, 2006), they were important here to enable systematic comparisons between readerships of different newspapers. We quantified our data in the form of the total number of comments per newspaper in which each theme appeared.

## 3 RESULTS

### 3.1 Moral engagement and disengagement strategies in the comments

We found evidence of several types of moral engagement and disengagement in the comments. Forty‐nine comments (16%) did not fit into Bandura's scheme. All remaining comments included material relevant to at least one locus of Bandura's theory, as described in Table 2. Note that total numbers of comments containing engagement or disengagement strategies are less than the sum of all strategies because some comments contained more than one strategy.

**Table 2:** Frequency of moral engagement and disengagement strategies by newspaper and overall

Moral stance	Locus and strategy	Examples	No. of comments in which this strategy was coded			
			Guardian	Mail	Telegraph	Total
Disengagement	1 (behaviour): Palliative comparison	There are many other threats to humans that are probably much more dangerous and something can be done in next 50 years. Boy who cries wolf distracts from real dangers. (Telegraph)	1	7	3	11
	1 (behaviour): Justify inaction (action on ACC as harmful)	I can't believe they're still at it with “the climate is changing—we're all going to die” … They are great at trying to induce guilt and panic—but have never explained why the destruction of western economies through ever more ridiculous CO2 “reductions” will help the poor who are, as the AGWers say, more at risk. […] (Telegraph)	1	0	3	4
	2 (agency). Diminished agency and/or responsibility	What I love is the hypocrisy of all this global warming cr*p! […] Then we poor old Brits have to lead the way! Hang on yet again! We produce less the 1% of global CO2 emissions! (Telegraph)	1	3	7	11
	3 (outcomes): Scepticism regarding (A)CC	The so called experts discount the real reason for our planets weather fluctuations and that is the Sun and its activity. The Sun goes through a cycle of Sun spots which heats up our Earth brining storms and drought to areas and then the Sun cools and thus the Earth cools bringing mini or long term Ice Ages and wet weather to the once dry zones. Our constantly changing weather has nothing to do with plant food, CO2, but all to do with the Sun's activity. (Mail)	24	73	77	174
	3 (outcomes): ACC has beneficial effects	What's this guy on about? The US had a massive corn harvest of 355 million metric tonnes in 2013. The Thai government has been overpaying for rice for the past two years as a populist policy and is sitting on a record 17 million tonne stockpile of the grain. Rice prices are set to plunge. Higher CO2 levels will mean greater crop yields, not lower. [...] (Telegraph)	4	3	2	9
	Generic assertions of nonengagement	They're still beating this drum and no one cares. […] (Mail)	0	5	5	10
	Any kind of disengagement		28	80	83	191
Engagement	1 (behaviour): Advocating action	Sad truth is we have known this for years and we have already squandered too much time. If we stopped all emissions now the temperature will continue to increase for decades to come because of what we have already done to the climate system. We cannot afford to delay urgent action any longer. This really is an emergency. I will gain no pleasure from history vindicating my views. A radical rethink about how we go about life on this planet is needed right now. Not tomorrow. Tomorrow is too late. (Guardian)	11	1	0	12
	3 (outcomes): Support regarding reality of ACC	Here's a challenge for all you deniers out there—what, if anything, would make you change your mind? I am firmly convinced by the scientific consensus, buttressed by basic physics … but I suppose there remains the 1% possibility that the theory is wrong. So here's my pledge—if at least 5 of the next 10 years are not the warmest yet recorded, I'll reconsider my position. What would make you reconsider yours? (Guardian)	42	6	8	56
	3 (outcomes): ACC has harmful effects	Sir,Ocean,food crops,melting glaciers are are affected by global warming.Everyone knows it. […] (Guardian)	19	2	3	24
	Any kind of engagement		51	8	10	69

Taking disengagement first, at Locus 1 (evaluation of behaviour or issue), 11 comments featured palliative comparison of ACC with other issues, such as population growth, and four comments justified inaction on the basis that action on ACC is harmful. At Locus 2 (agency), 11 comments argued that their own or their in‐group's agency was limited. Locus 3 (outcomes) was by far the most popular site of disengagement, with nine comments asserting that ACC has beneficial effects and 174 negating the existence of ACC. There were no comments denigrating victims (Locus 4).

Turning to moral engagement, at Locus 1, 12 comments asserted the need for action. Again, Locus 3 (outcomes) was most prevalent, with 56 comments asserting the reality of ACC, and 24 specifying negative effects. Loci 2 (emphasising agency) and 4 (value victims) were not evident. See Table 3 for an overview of the frequency of each locus by newspaper.

**Table 3:** Frequency of moral engagement and disengagement loci by newspaper

Locus	Guardian		Mail		Telegraph		Total	
	Disengagement	Engagement	Disengagement	Engagement	Disengagement	Engagement	Disengagement	Engagement
1: Behaviour	2	11	7	1	6	0	15	12
2: Agency	1	0	3	0	7	0	11	0
3: Outcomes	28	51	74	8	77	10	179	69

### 3.2 Differences between newspapers

To assess whether the strategies employed by commenters varied according to the newspaper they were commenting on, a series of chi‐square goodness of fit analyses were conducted. Strategies were only analysed if they appeared in more than 10 comments. Where expected cell frequencies fell below five, exact tests were used.

Our hypotheses were supported. There were significant differences between newspapers for all moral disengagement strategies that were tested; namely, palliative comparison, χ^2^(2) = 5.091, exact *p* = .045; diminishing agency, χ^2^(2) = 5.091, exact *p* = .045; and scepticism, χ^2^(2) = 30.034, *p* < .001. There was also a significant difference in the prevalence of any form of moral disengagement, χ^2^(2) = 30.042, *p* < .001. In all cases, disengagement strategies were more common in the right wing Daily Mail and Telegraph (appearing in 82% of all comments) than they were in the Guardian (28% of comments).

For moral engagement, there were significant differences between newspapers for all strategies: advocating action, χ^2^(1) = 8.333, exact *p* = .006; support for the reality of ACC, χ^2^(2) = 43.857, *p* < .001; and claims that ACC has harmful effects, χ^2^(2) = 22.750, *p* < .001. There was also a significant difference in the prevalence of any moral engagement strategy, χ^2^(2) = 51.217, *p* < .001. In all cases, the strategies were more common in left wing than right wing newspapers. Just over 50% of Guardian comments included moral engagement, compared with 9% of comments on the right wing papers.

Our hypotheses regarding the relative frequencies of moral engagement and disengagement in the comments on each newspaper were also supported. Engagement was significantly more frequent than disengagement for comments on the Guardian, χ^2^(1) = 6.696, *p* = .010; the opposite was true for the Daily Mail, χ^2^(1) = 58.909, *p* < .001, and Telegraph, χ^2^(1) = 57.301, *p* < .001.

We also analysed the moral content of the five comments in our sample, which were most highly recommended by other readers for each newspaper. For the Mail, the total number of recommendations for the top five comments was 3,868. All five comments were coded as moral disengagement. For the Telegraph, there were 283 recommendations for the top five comments, and again, all five were coded as moral disengagement. For the Guardian, there were 181 recommendations for the top five comments, of which three included moral engagement, one moral disengagement, and one was coded as “other.”

### 3.3 Presence of moral themes not captured by Bandura's scheme

During the initial round of broad coding, all authors were independently struck by a subsection of the many comments negating the existence of ACC. Sceptical truth claims were constructed in several different ways, including ACC as an extremist religion (“green religious zealots”), an overreaction (“screaming, idiotic hysteria”), bad science (“those wonky academics who had climbed up on the ‘Global Warming’ bandwagon and claimed it was ‘settled science’”), or simply wrong (“global warming garbage”). Of significance here was an additional, highly moralised set of claims about dishonesty. Specifically, many comments described ACC proponents as deliberately deceitful, having ulterior motives (particularly financial), using ACC as an excuse for taxation and/or control, having vested interests, and/or being engaged in a conspiracy, as illustrated in the following examples:

Another chapter in the Big Con Job no doubt rolled out to keep all the boffin's in employment wined and dined in flash hotels and first class travel at our expense. (Mail)

Sick of this garbage they keep shoving down our throats. CC is absolute rubbish, at least as far as humans having anything to do with it. this is nothing more than a ways and means to control us and tax us further. (Mail)

Pathetic. Everyone knows that “climate change” was invented by politicians (particularly Labour ones) to give themselves a “reason” for big tax rises. (Telegraph)

Ah … the annual stirring of the pot to keep the funds flowing. […] (Telegraph)

These comments all undermine the moral integrity of ACC proponents, who are constructed as deceptive (“Big Con Job”), driven by political (“a ways and means to control us”) and financial (“the annual stirring of the pot to keep the funds flowing”; “a ‘reason’ for big tax rises”) ulterior motives. The dishonesty theme directed toward ACC proponents appeared in four Guardian comments, 34 Mail comments, and 39 Telegraph comments, a statistically significant difference, χ^2^(2) = 27.922, *p* < .001. In contrast, claims that sceptics were dishonest appeared in only four comments, all in the Guardian.

## 4 DISCUSSION

Although the morality of ACC has been widely acknowledged as important, the current study is the first to examine whether and how the public brings morality to bear on discussions about ACC. We found that the majority of comments employed strategies outlined in our extension of Bandura's theory to morally engage or disengage with ACC as a harm/care moral issue. The prevalence of relevant material suggests that the theory offers a useful framework for understanding how moral concerns around ACC are expressed or averted in practice.

Not all of the loci of Bandura's theory were represented in the comments. The most ubiquitous, for both engagement and disengagement, was the third locus, focusing on outcomes. Loci 1 (behaviour) and 2 (agency) appeared relatively infrequently, and Locus 4, focused on victims, did not appear at all. This is surprising in that Laksa (2014) found references to in‐group and out‐group victims of ACC in British newspapers. However, claims of harmful effects usually specified (to some extent) whom the effect was on, so one might argue that moral engagement at Locus 4 tends to arise out of engagement at Locus 3, rather than being raised as an issue in its own right.

The focus on outcomes that we found for both engagement and disengagement is unsurprising in light of previous research finding that ACC is usually framed as a harm/care issue (Gardiner, 2006; Hansen, 2010; Markowitz, 2012), and given that outcomes were emphasised in the newspaper articles that commenters were responding to. The current study adds to the literature by showing that the issue of outcomes resonates with the general public, and that harm/care is an important moral foundation upon which public discourse around ACC is based.

Although our analysis demonstrates the usefulness of Bandura's (2007) framework for understanding ACC moralising, it also finds it wanting, in that any resistance to embracing and responding to ACC as a harm‐based moral issue is automatically classified by Bandura's theory as disengagement, and thus as immoral or amoral. This problem manifested itself in two ways in our findings.

First, at Locus 1, Bandura counts as disengagement any assertions that diminish the gravity of the issue at stake (Bandura, 1999, 2007). A small number of comments in our dataset achieved this through palliative comparison of ACC with other issues. Although they might indeed disengage from ACC, such comments tended to do so by morally engaging with another issue. This shift from one moral issue to another might be better described as prioritisation rather than disengagement. There is evidence that people subscribe to several moral values in principle but often prioritise one over another in practice (Woods, 2013; Turiel, Killen, & Helwig, 1987). Therefore, a person's moral disengagement from ACC can be better understood if contextualised by their engagement with other, potentially conflicting moral values.

Second, a strong theme running through the comments was a critique of the integrity of ACC supporters, who were constructed as dishonest, unscrupulous, and untrustworthy. This dishonesty theme represented one of several ways in which the credibility of ACC proponents was undermined. Bandura (2007) argues that such undermining is a type of scepticism, which is in turn one form of moral disengagement operating at the third locus, outcomes. Thus, using Bandura's framework, comments accusing supporters of dishonesty appear as disengagement. However, our analysis suggests that many sceptics experience their critique of ACC as a strong *engagement* with a different set of moral issues, revolving around trust, honesty, and integrity. We suggest that this deception theme could be grounded in at least four of Haidt's moral foundations: fairness (ACC supporters as unfairly cheating and exploiting the populace), loyalty (supporters as betraying the populace), sanctity (supporters as having impure motives), and liberty (governments as oppressive; Haidt & Joseph, 2008; Iyer et al., 2012).

The dishonesty theme was quite widespread, appearing in 26% of all comments. It is a discourse with a considerable history, which may have started with a piece in the Wall Street Journal in 1996, and gained traction through elite anti‐environmentalist groups and so‐called “Climategate” (Goertzel, 2010; Nerlich, 2010). It has continued to appear in the media and online commentary (Jaspal et al., 2013; Koteyko et al., 2013; Nerlich & Koteyko, 2009) and was also regularly asserted by Donald Trump prior to his presidency, perhaps informing his decision to withdraw the USA from the Paris Agreement (Baker, 2017). These studies, along with our findings, suggest that the construction of ACC proponents as deliberately deceptive and corrupt already has considerable momentum in the public sphere.

What the “ACC proponents as dishonest” discourse and “ACC is less important than X” claims have in common is that they both appear purely as moral disengagement in Bandura's theory but can be seen as moral engagement when placed in the context of other, potentially competing, moral concerns. There is a need, then, for a theory that can capture a wider range of moralising around ACC than that encapsulated in Bandura's theory, in order to gain a fuller understanding of the moral reasoning circulating among lay climate sceptics.

One way of proceeding would be to view Bandura's theory as one of several possible moral landscapes on ACC, each with its own areas of concern and sensitivity. We suggest that each person constructs their own moral landscape discursively in collaboration with valued others, leading different groups of people to interpret and react to the same events (such as the release of an IPCC report) in radically different ways. This possibility could be explored by examining how members of distinct groups within society construct and moralise ACC. Such research would require a bottom‐up emphasis, focusing on what people do and say in practice, and not be constrained by current theorising. For instance, not only is the “dishonesty” theme identified in the current research not captured by Bandura's theory, it also does not obviously or neatly fit into any single moral foundation outlined by Haidt and Joseph (2008) and hence might be lost or distorted if moral landscape exploration were theory‐dominated. Discursive psychology (Edwards & Potter, 1992) and social representations theory (Moscovici, 2000; Voelklein & Howarth, 2005) may offer useful ways to make sense of how these moral landscapes emerge as people seek to form, articulate, and defend particular claims about ACC in relation to those around them.

The articulation of moral landscapes has the potential to provide a fuller understanding of ACC scepticism and/or inaction and could help to ensure that efforts to persuade and explain are accessible to their audience (Feinberg & Willer, 2015). For instance, the dishonesty theme enables sceptics to dismiss ACC truth claims, and their harm/care‐based moral implications, without directly engaging with those claims, but instead by questioning the credibility of those who make the claims. Efforts to persuade based on truth claims seem unlikely to succeed without also addressing the moral concerns around deception and trust. Furthermore, analyses of how moral landscapes are constructed by members of a particular community may reveal points of weakness or dissent, where views may be relatively amenable to constructive dialogue and persuasion. For instance, although many participants in online Daily Mail discussions treat climate scientists with distrust, they may perhaps construct scientists in other fields, such as cancer research or astronomy, differently. Such instability around the moral valuing of scientists might represent opportunities to shift the moral landscape regarding climate scientists. This hypothetical example demonstrates the potential utility of a rich understanding of a person's moral landscape, and how this is constructed and maintained dialogically.

Such an analysis would need to take into account the way in which moral landscapes are organised around political allegiance. As hypothesised, we found that comments on right wing newspapers contained significantly more moral disengagement than those on left wing newspapers, whereas the opposite was true for moral engagement. Moreover, there was significantly more moral engagement than disengagement in the Guardian and significantly more disengagement than engagement in the Mail and Telegraph. Our findings are novel in that they demonstrate that the differential moral engagement and disengagement (Carvalho, 2007; Carvalho & Burgess, 2005; Doulton & Brown, 2009; Laksa, 2014; Painter & Gavin, 2015; Woods et al. 2012) by the left‐ and right‐wing media in Britain also extend to their readers. Rates of engagement and disengagement were similar for the two right wing papers, suggesting that those who comment on them share a moral perspective that cuts across the different social status of the tabloid—broadsheet divide (Chan & Goldthorpe, 2007).

We also found that the dishonesty theme was significantly more widespread in comments on the right wing than left wing newspapers. Again, it was popular in both the Mail and Telegraph, suggesting broad appeal. Koteyko et al. (2013) argue that the construction of ACC proponents as dishonest maps onto a long‐standing tabloid newspaper frame associating politics with corruption, greed, and dishonesty. This frame may link to a “conspiracy” mindset, which views anything asserted by the establishment as suspect (Goertzel, 2010). Of the three newspaper articles used in the current study, only the Daily Mail article referred to governments “lobbying” for a change in the wording of the report, thus potentially opening the door to speculations around the influence of political interests in scientific reporting. However, the dishonesty theme was even more common in comments on the broadsheet Telegraph. Therefore, either the “corruption” discourse identified by Koteyko et al. (2013) in the tabloids also extends to broadsheet readers, and/or the dishonesty discourse can arise from other sources, such as free market ideologies, which are related to low environmental concern (Heath & Gifford, 2006; Pidgeon, 2012), and which may be popular among conservative middle class readers.

This study demonstrates that readers' online comments represent a rich source of data on public discourse, enabling us to move beyond analyses of the media, toward how media claims are interpreted and mobilised by the general public. Although we do not yet know how representative online comments are of the views of the entire readership of particular newspapers, the large numbers of recommendations that some comments received, particularly in the Daily Mail, suggest that the views expressed do resonate to some degree with the readership, beyond those who post comments.

The relationship between media claims and the beliefs and assertions of readers is not a simple one, and our understanding of it remains in its infancy. For instance, Gaskell, Bauer, Durant, and Allum (1999) found that in the case of food biotechnology, it was the coverage of scientific controversies rather than positive or negative coverage per se that predicted more negative public perceptions of genetically modified food. Gaskell et al. (1999) conclude that “Different histories of media coverage and regulations go together with different public perceptions, and these in turn reflect deeper cultural sensitivities” (p. 385). Therefore, rather than providing evidence of the direct effects of media coverage on people's perceptions of social realities, analysing readers' comments gives a unique opportunity to witness their sense‐making activities. This sense‐making process may constitute collective symbolic coping (Wagner & Kronberger, 2001), that is, “the collective activity of a group struggling to maintain the integrity of its worldview which is also crucial for social identity” (p. 4)—an identity that is to some extent constituted through people's relationships with particular newspapers as broadsheets or tabloids; left or right wing. Thus, although acknowledging that these sense‐making practices might be specific to the characteristics of online commenters (see Martin, 2016, for an interesting critical approach to understanding barriers and facilitators to commenting online), they might offer insight into the processes underlying this important activity and help developing testable theoretical frameworks for future research.

To conclude, this study has shown that in online commentary, people do in practice employ a range of the moral disengagement strategies outlined by Bandura (1999, 2007) and Bandura et al. (1996), and their engagement corollaries, particularly pertaining to the impacts of ACC. Moreover, these strategies were highly polarised along political lines, such that readers of left wing media expressed more engagement with the morality of harm/care around ACC than did readers of more right wing media. We also found that readers of right wing media articulated a set of moral concerns around dishonesty, which were not adequately captured by Bandura's framework. We suggest that people's moral orientations toward ACC may be best understood as part of a broader moral landscape, defined as the set of moral significations which each person constructs dialogically, and through which they interpret the people and events around them. Understanding how members of particular communities are constructing their moral landscapes may aid the development of effective routes to persuasion and reassurance.
